# Public Knowledge and Management Practices of Anaphylaxis in Al-Ahsa, Saudi Arabia: A Cross-Sectional Study

**DOI:** 10.7759/cureus.77818

**Published:** 2025-01-22

**Authors:** Aminah Alhumam, Abdulaziz S Hadadi, Mohammed I Al Fehaid, Abdulaziz K Alsubaie, Khalid J Alqadhib, Ahmed K Alnawah, Saud K Alsubaie

**Affiliations:** 1 Dermatology, King Faisal University, Al-Ahsa, SAU; 2 Medicine, King Faisal University, Al-Ahsa, SAU

**Keywords:** anaphylaxis, emergency response, epinephrine, knowledge, saudi arabia

## Abstract

Introduction

Anaphylaxis is a life-threatening allergic reaction that has a rapid onset and the potential for fatal outcomes. In Saudi Arabia, anaphylaxis has become more prevalent, particularly among children, yet the knowledge of underlying possible causes and pathogenesis and its management remains insufficient. This study aims to assess the level of awareness and management practices of anaphylaxis among residents of Al-Ahsa, Saudi Arabia.

Methods

A descriptive cross-sectional study was conducted from May to June 2024 among 384 participants in Al-Ahsa. Data were collected using an online, self-administered questionnaire distributed via social media platforms. We excluded those who refused to participate, those who were healthcare professionals, and those residing outside Al-Ahsa and were non-Saudi. The questionnaire comprised 21 items evaluating demographic characteristics and knowledge of anaphylaxis, including its clinical features, causes, risk factors, emergency actions, previous experiences, and appropriate responses to anaphylaxis signs.

Results

The study revealed that most participants (154, 40%) were aged 21-30 years, 234 (61%) were male, and 230 (60%) had a university education. Awareness of anaphylaxis was relatively high, with 271 participants (71%) reporting they had heard of the condition. Additionally, 221 participants (58%) recognized its potentially fatal nature. Knowledge of common triggers, such as food and insect stings, was high, with 344-366 participants (90-95%) identifying these as causes of anaphylaxis. However, recognition of less common triggers like latex was lower, with only 185 participants (48%) aware of this risk. When it came to treatment, just 71 participants (18%) correctly identified epinephrine as the first-line treatment for anaphylaxis. Although 160 participants (40%) indicated they would seek hospital care in an emergency, only 28 (7%) recognized the need to administer epinephrine. Overall, 245 participants (64%) demonstrated good knowledge of anaphylaxis. Significant differences in knowledge levels were observed based on gender (p = 0.017), marital status (p = 0.037), education level (p = 0.049), prior awareness of anaphylaxis (p = 0.002), and having witnessed an anaphylaxis case before (p = 0.007).

Conclusion

Awareness of proper anaphylaxis and allergy management, particularly the use of epinephrine, is notably lacking. Targeting public health awareness is essential to enhance public knowledge and preparedness for managing anaphylactic emergencies in Al-Ahsa, Saudi Arabia.

## Introduction

Anaphylaxis is defined as a severe, life-threatening systemic allergic reaction that typically has a rapid onset, which can impair the respiratory or circulatory systems [1]. The World Allergy Organization defines it as “typical skin symptoms” AND “significant symptoms from at least one other organ system” OR “exposure to a known or probable allergen for that patient, with respiratory and/or cardiovascular compromise” [2]. Clinically, anaphylaxis most often presents with urticaria, angioedema, erythema, pruritus, respiratory distress, tongue swelling, and, in some cases, death due to cardiovascular collapse or airway obstruction [3].

In Saudi Arabia, the prevalence of anaphylaxis among emergency department (ED) admissions was reported as 0.00026%, with 60.9% of cases occurring in pediatric patients (ages one to 16 years) and 39.1% in adults (ages 17-40 years) [4]. In the United States, anaphylaxis occurs annually in 30 per 100,000 people, with a reported mortality of 1-2% [5]. Furthermore, recent studies indicate an increasing incidence and prevalence of anaphylaxis over the past two decades, possibly due to a rise in allergic sensitization to foods, especially among children, increased outdoor activities, and the broader use of biological medications [6]. The primary triggers of anaphylaxis across all age groups include ingested foods, insect stings, and medications, while less common triggers involve cats, latex, cleaning agents, environmental allergens, and exercise [7].

Given the rapid progression of anaphylaxis, prompt recognition and timely intervention are essential to prevent fatal outcomes [8]. Laypersons are pivotal in managing anaphylactic reactions, as most occur outside healthcare settings [9]. However, a study in Turkey concluded that the parents of children who experienced life-threatening anaphylaxis have no sufficient level of awareness [10]. Another study among the general population in the western region of Saudi Arabia reported that only 53% of participants could identify multiple symptoms accurately. In comparison, 38.9% were uncertain about the proper diagnostic methods. Moreover, only 18.4% recognized epinephrine as the first-line treatment, 25% knew the correct administration route (intramuscular), and 43.1% were aware of the need to visit the ER after using epinephrine [11]. These findings suggest that inadequate knowledge of anaphylaxis management, compounded by the absence of formal training and educational programs on allergic emergencies, significantly limits effective response to anaphylactic events. Consequently, educating patients and caregivers about the prevention, recognition, and treatment of anaphylaxis is vital [12]. There is a lack of studies in our region (Al-Ahsa, Saudi Arabia) that address anaphylaxis, particularly in the context of differing cultural and lifestyle factors. These unique regional characteristics may contribute to heightened food sensitivities and an increased prevalence of prophylactic reactions. Highlighting these gaps underscores the urgent need for research and targeted educational programs to address anaphylaxis awareness and management within this cultural context.

## Materials and methods

Study design

A descriptive cross-sectional study was conducted between May and June 2024 among the general population in Al-Ahsa, located in the Eastern Province, Kingdom of Saudi Arabia, excluding those who refused to participate, those who were healthcare professionals, and those residing outside Al-Ahsa and were non-Saudi.

Sample size

The sample size was determined using Raosoft software (Raosoft Inc., Seattle, WA). Considering a 50% predicted frequency with a margin of error of 5% and a 95% confidence interval, the sample size was calculated to be 384.

Data collection

Using a non-probability convenient sampling technique, an online self-administered questionnaire was used. The questionnaire was formulated from Albalawi et al.’s study [13] after an extensive literature review. It was translated into Arabic, the major language in the country, and sent in Google Forms (Google, Inc., Mountain View, CA) to be distributed through social media platforms, including Telegram (Pavel Durov and Nikolai Durov, Dubai, UAE), X (formerly Twitter, X Corp., Bastrop, TX), WhatsApp (Meta Platforms, Menlo Park, CA), and others, and by direct contact. To ensure the face validity of the questionnaire, a group of experts revised it. Consequently, a pilot study among 30 participants was conducted, and the feedback from the pilot study was used to improve the clarity and comprehensibility of the questionnaire. The responses from the pilot study were not included in the final analysis.

The questionnaire has been structured into four sections. The first section gathers demographic information of the participants, including age, marital status, occupation, education, and the number of children.

The second section assesses the respondents' knowledge regarding anaphylaxis using 21 questions, including clinical features of anaphylactic reactions, causes, risk management, action in an emergency, and previous experiences. The third section includes the participants' knowledge of reported symptoms of anaphylaxis. In addition, the fourth section focuses on the right action when noticing signs of anaphylaxis reported by residents of Al-Ahsa, Saudi Arabia.

Statistical analysis

The data were cleaned in an Excel sheet (Microsoft® Corp., Redmond, WA) and imported to SPSS software version 27 (IBM SPSS Statistics for Windows, IBM Corp., Armonk, NY). To calculate the knowledge score, the correct answers were coded as one, while incorrect and not-sure answers were coded as zero. The normality of the score was tested using a histogram and Kolmogorov-Smirnov test, and participants who scored ≥ the median were considered to have "good knowledge." Descriptive statistics were used to calculate the median and interquartile range for the continuous variables and frequencies with percentages for categorical variables. Pearson's chi-squared and Fisher's exact tests were used to identify determinants of anaphylaxis knowledge. Kruskal-Wallis and Wilcoxon rank-sum tests were used to determine the associated demographic characteristics and the knowledge of anaphylaxis among residents in Al-Ahsa, Saudi Arabia. A p-value of <0.05 was considered significant for this study.

Ethical consideration

The study followed the Declaration of Helsinki for human research, and full approval was obtained from the committee of ethics of King Faisal University perceived from the Deanship of Scientific Research (KFU-REC-2023-MAY-ETHICS869). Written informed consent was obtained from participants, and they will not participate until they give full approval. The participants' privacy and confidentiality was maintained, and their information was used for scientific purposes only.

## Results

Table 1 provides an overview of the demographic characteristics of the 384 participants included in the study. The majority, 154 participants (40%), were aged 21-30 years, followed by 130 participants (34%) aged 15-20 years. Participants aged 41 years and above accounted for 50 (13%), while those aged 31-40 years comprised 46 (12%). In terms of gender distribution, 234 participants (61%) were male, and 150 participants (39%) were female. Marital status showed that more than half, 265 participants (69%), were unmarried, while 119 participants (31%) were married. Regarding the number of children, the majority, 277 participants (72%), had no children, followed by 65 participants (17%) with two to four children, 24 participants (6.3%) with five or more children, and 18 participants (4.7%) with one child. For education level, 230 participants (60%) had completed university education, while 154 participants (40%) had primary, intermediate, or secondary education. Only one participant (0.3%) was illiterate. Employment status revealed that 241 participants (63%) were not working, 127 participants (33%) were working full-time, and 14 participants (3.6%) were working part-time.

**Table 1:** Demographic characteristics of study participants (N = 384)

Study data	Frequency (percentage)
Age	
15-20 years	132 (34%)
21-30 years	154 (40%)
31-40 years	47 (12%)
41 years and above	51 (13%)
Gender	
Female	149 (39%)
Male	235 (61%)
Marital status	
Married	119 (31%)
Unmarried	265 (69%)
Number of children	
Zero	276 (72%)
One	18 (4.7%)
Two to four	66 (17%)
Five	24 (6.3%)
Education	
Illiterate	1 (0.3%)
Primary/intermediate/secondary	154 (40%)
University	229 (60%)
Occupation	
Not working	242 (63%)
Working full time	128 (33%)
Working part-time	14 (3.6%)

Table 2 shows that 71% (271) of participants reported having heard about anaphylaxis, while 21% (81) had witnessed a case of anaphylaxis. Regarding triggers of anaphylaxis, 95% (366) were aware that certain foods could cause anaphylaxis, while 1.3% (five) disagreed, and 3.4% (13) did not know. Similarly, 90% (344) recognized some drugs as potential causes, while 2.3% (nine) disagreed, and 8.1% (31) did not know. Additionally, 91% (350) acknowledged animal or insect bites as triggers, while 3.1% (12) disagreed, and 5.7% (22 did not know. Dust or pollen was identified as a cause by 86% (332) of participants, while 3.9% (15) disagreed, and 9.6% (37) did not know. Only 23% (87) agreed that exercising is a trigger, whereas 43% (167) disagreed, and 34% (130) did not know. Similarly, 48% (185) recognized latex as a cause of anaphylaxis, while 13% (49) disagreed, and 39% (150) did not know. More than half of the participants, 58% (221), believed anaphylaxis could lead to death, while 9.1% (35) disagreed, and 33% (128) did not know. Similarly, 58% (221) acknowledged that previous anaphylaxis increases the risk of another attack, while 6% (23) disagreed, and 36% (140) did not know. Furthermore, 49% (190) believed asthma increases the risk of anaphylaxis, whereas 9.9% (38) disagreed, and 41% (156) did not know. In terms of treatment, 37% (141) incorrectly believed that anaphylaxis is best treated with an antihistamine injection, while only 18% (71) correctly identified epinephrine injection as the best treatment. Additionally, 4.9% (19) believed anaphylaxis had no treatment, 37% (143) did not know the treatment, and 2.6% (10) considered it a self-limited condition.

**Table 2:** Knowledge of anaphylaxis among Al-Ahsa residents (N = 384) *Data presented as frequency (percentage)

Characteristic	No*	Yes*	I do not know
Heard about anaphylaxis before	113 (29%)	271 (71%)	0
Witnessed a case of anaphylaxis before	303 (79%)	81 (21%)	0
Some foods can cause anaphylaxis	5 (1.3%)	366 (95%)	13 (3.4%)
Some drugs can cause anaphylaxis	9 (2.3%)	344 (90%)	31 (8.1%)
Animals or insect bites can cause anaphylaxis	12 (3.1%)	350 (91%)	22 (5.7%)
Dust or pollen can cause anaphylaxis	15 (3.9%)	332 (86%)	37 (9.6%)
Exercising can cause anaphylaxis	167 (43%)	87 (23%)	130 (34%)
Latex can cause anaphylaxis	49 (13%)	185 (48%)	150 (39%)
Anaphylaxis can lead to death	35 (9.1%)	221 (58%)	128 (33%)
Previous anaphylaxis increases the risk of another attack	23 (6.0%)	221 (58%)	140 (36%)
Having asthma puts the person at higher risk of anaphylaxis	38 (9.9%)	190 (49%)	156 (41%)
Anaphylaxis is			
Best treated with an antihistamine injection	141 (37%)	0	0
Best treated with epinephrine injection	71 (18%)	0	0
Has no treatment	19 (4.9%)	0	0
I do not know	143 (37%)	0	0
Self-limited condition	10 (2.6%)	0	0

**Figure 1:**
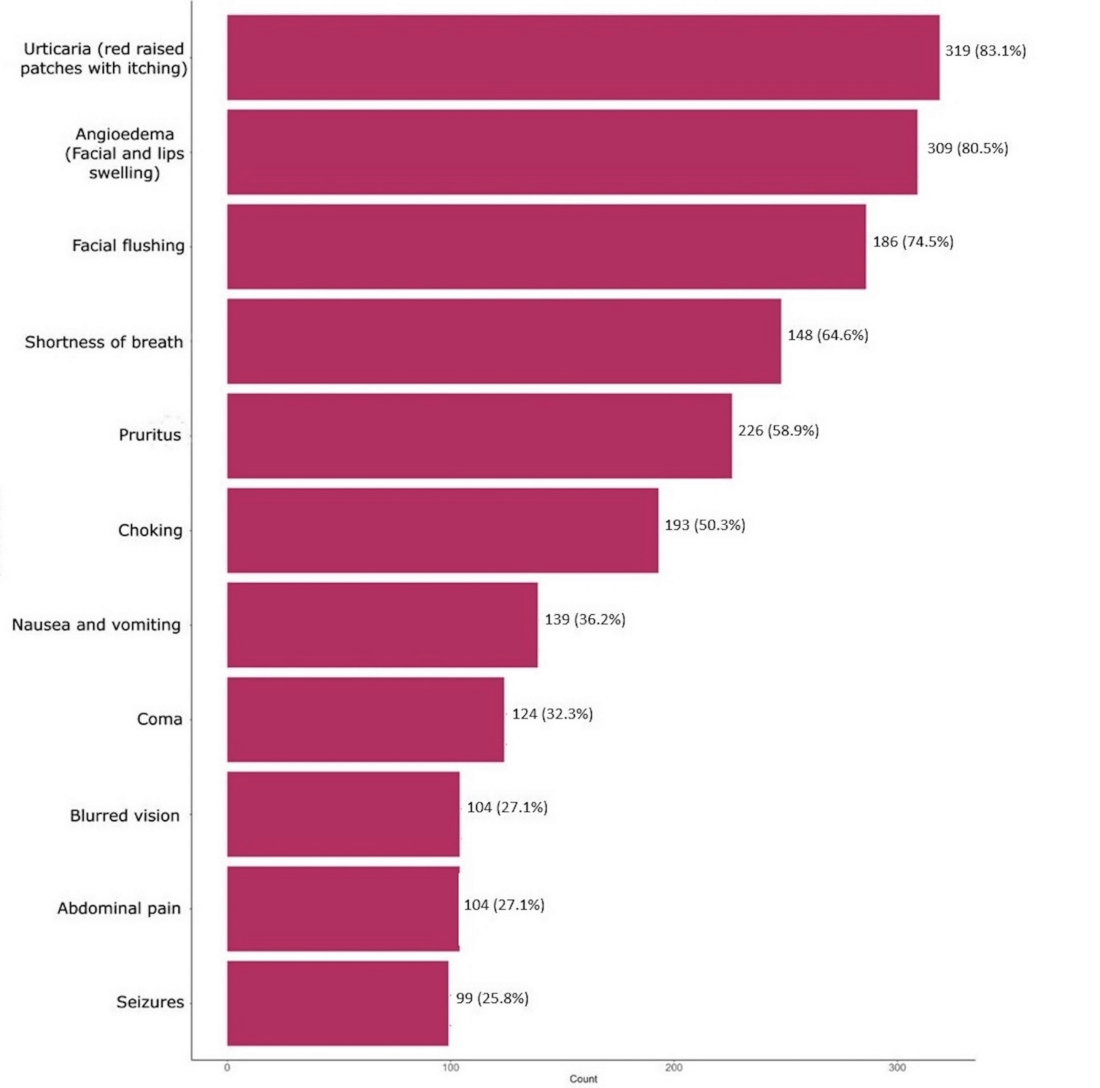
Knowledge about symptoms of anaphylaxis among Al-Ahsa residents

Figure 1 illustrates the recognition of symptoms associated with anaphylaxis among participants. Nearly 80% (324, 83.1%) identified urticaria (red raised patches with itching) as the most commonly reported symptom, while 314 (80.5%) recognized angioedema (facial and lip swelling). Facial flushing and shortness of breath were acknowledged by 291 (74.5%) and 252 (64.6%) participants, respectively. Pruritus and choking were identified by 230 (58.9%) and 196 (50.3%) respondents, respectively. Symptoms such as nausea and vomiting were recognized by 142 (36.2%) participants, while coma was noted by 127 (32.3%). Blurred vision and abdominal pain were each identified by 107 (27.1%), and seizures were the least recognized symptom, reported by 102 (25.8%) respondents.

Table 3 highlights the distribution of knowledge about anaphylaxis among participants. More than half of the respondents, 245 (63.8%), demonstrated good knowledge of anaphylaxis. Knowledge levels significantly differed by gender (p = 0.017), with males showing lower levels of knowledge compared to females. Marital status also played a role in knowledge levels, as married individuals exhibited higher levels of awareness than their unmarried counterparts (p = 0.037). Similarly, education level was a significant factor (p = 0.049), with participants who had completed university education demonstrating better knowledge than those with lower educational attainment. Furthermore, participants who had previously heard about anaphylaxis (p = 0.002) or witnessed a case of anaphylaxis (p = 0.007) were more likely to have good knowledge about the condition.

**Table 3:** Determinants of knowledge of anaphylaxis among Al-Ahsa residents *Data presented as frequency (percentage) **Pearson's chi-squared test; Fisher's exact test

Characteristic	Poor knowledge (N = 139)*	Good knowledge (N = 245)*	p-value^**^
Age			
15-20 years	54 (39%)	78 (32%)	0.3
21-30 years	55 (40%)	99 (40%)	
31-40 years	12 (8.6%)	35 (14%)	
41 years and above	18 (13%)	33 (13%)	
Gender			
Female	43 (31%)	106 (43%)	0.017
Male	96 (69%)	139 (57%)	
Marital status			
Married	34 (24%)	85 (35%)	0.037
Unmarried	105 (76%)	160 (65%)	
Number of children			
Zero	108 (78%)	168 (69%)	0.086
One	6 (4.3%)	12 (4.9%)	
Two to four	15 (11%)	51 (21%)	
Five or more	10 (7.2%)	14 (5.7%)	
Education			
Illiterate	1 (0.7%)	0 (0%)	0.049
Primary/intermediate/secondary	64 (46%)	90 (37%)	
University	74 (53%)	155 (63%)	
Occupation			
Not working	92 (66%)	150 (61%)	0.5
Working full time	41 (29%)	87 (36%)	
Working part-time	6 (4.3%)	8 (3.3%)	
Heard about anaphylaxis before			
No	54 (39%)	59 (24%)	0.002
Yes	85 (61%)	186 (76%)	
Witnessed a case of anaphylaxis before			
No	120 (86%)	183 (75%)	0.007
Yes	19 (14%)	62 (25%)	

Table 4 shows participants who had heard of anaphylaxis before and those who had witnessed an anaphylaxis case scored higher on the knowledge scale (p < 0.001 and p = 0.013, respectively). The median knowledge scores showed that females, those with higher education, and those with prior exposure to anaphylaxis information had slightly higher knowledge scores.

**Table 4:** Association between demographic characteristics and the knowledge of anaphylaxis among Al-Ahsa residents *Knowledge score: median (IQR) **Kruskal-Wallis rank-sum test; Wilcoxon rank sum-test

Characteristic	N = 384^*^	p-value^**^
Age		
15-20 years	6.00 (5.00, 7.00)	0.3
21-30 years	6.00 (5.00, 8.00)	
31-40 years	6.00 (5.50, 7.50)	
41 years and above	6.00 (5.00, 7.00)	
Gender		
Female	7.00 (5.00, 8.00)	0.023
Male	6.00 (5.00, 7.50)	
Marital status		
Married	6.00 (5.00, 8.00)	0.12
Unmarried	6.00 (5.00, 8.00)	
Number of children		
Zero	6.00 (5.00, 8.00)	0.2
One	7.00 (5.00, 7.75)	
Two to four	7.00 (6.00, 7.00)	
Five	6.00 (5.00, 8.00)	
Education		
Illiterate	3.00 (3.00, 3.00)	0.010
Primary/intermediate/secondary	6.00 (5.00, 7.00)	
University	6.00 (5.00, 8.00)	
Occupation		
Not working	6.00 (5.00, 7.00)	0.2
Working full time	6.00 (5.00, 8.00)	
Working part-time	6.00 (4.25, 7.00)	
Heard about anaphylaxis before		
No	6.00 (5.00, 7.00)	<0.001
Yes	7.00 (5.00, 8.00)	
Witnessed a case of anaphylaxis before		
No	6.00 (5.00, 8.00)	0.013
Yes	7.00 (6.00, 8.00)	

Table 5 outlines the participants' responses to handling an anaphylaxis case. The most common response, reported by 160 participants (40%), was to go to the hospital. This was followed by 80 participants (20%) who would visit a primary healthcare center or a specialist and 60 participants (15%) who would call an ambulance. Fewer participants (28, 7%) recognized the need to administer epinephrine, while only nine (2.3%) mentioned antihistamines as a response. Notably, 36 participants (8.9%) admitted they did not know the correct course of action.

**Table 5:** Right action when noticing signs of anaphylaxis reported by Al-Ahsa residents (N = 384)

Characteristic	Frequency (percentage)
Epinephrine injection	27 (7.0%)
Call ambulance	58 (15%)
Go to the hospital	153 (40%)
Avoid triggers	28 (7.3%)
Go to the primary healthcare center/specialist doctor	75 (20%)
Antihistamine use	9 (2.3%)
I do not know	34 (8.9%)

## Discussion

This study, which aimed to assess the knowledge and behavior of the general population toward anaphylaxis, included 384 participants, with the majority in the age group 21-30 years; 60% were educated to university level, while only 0.3% were illiterate.

When their knowledge about anaphylaxis was assessed, 61% of the study participants had heard of anaphylaxis; only 21% had witnessed a case. Regarding their knowledge about possible triggers of anaphylaxis, certain foods were identified as a trigger by 95%, animal or insect bites were reported by 91%, and 90% identified that certain drugs could be triggers for anaphylaxis. In Tabuk, Saudi Arabia, anaphylaxis knowledge was assessed among parents, and similar results were obtained; 80% reported hearing about anaphylaxis, yet only 37% witnessed an anaphylaxis case. Again, food and drugs were identified as the major cause of anaphylaxis by the parents, followed by animal and insect bites [13].

Regarding anaphylaxis severity, more than half believed it could lead to death, and a similar percentage indicated that previous attacks increased the risk. Asthma was also identified as a potential risk factor. Regarding anaphylaxis management, the general population's knowledge was poor. More than a third believed that anaphylaxis could be treated with antihistamine injections, while only 18% correctly identified epinephrine injection as the best treatment. Parents’ knowledge about anaphylaxis in Tabuk reported that less than half agreed that previous attacks and asthma are risk factors for anaphylaxis. Only 3% were aware of using epinephrine as a treatment for anaphylaxis [13,14]. In Spanish communities, teachers expressed that they would not know how to respond to anaphylaxis or how to administer the necessary medication properly [15,16]. A similar assessment occurred in Qatar, where schools and families were assessed regarding their knowledge of anaphylaxis and its management. There was a miscommunication between families of children with allergies and the schools, where only 23% of families informed the school about their child's allergy. The majority in both communities could not recognize anaphylaxis symptoms, and only a few used EpiPen at the level of both families and schools [17]. Saudi teachers' understanding and behavior toward anaphylaxis were assessed by Alsuhaibani et al. The majority exhibited a low level of knowledge about anaphylaxis, and their practical understanding was generally low to moderate; however, they demonstrated a positive attitude toward the disease. The bulk of their information about anaphylaxis was from social media. The knowledge of Saudi teachers about food allergy specifically was assessed in a different study in the Jazan region. The authors conclude that teachers have poor knowledge about food allergy and minimal appreciation of the seriousness of its consequences; however, female teachers have higher overall knowledge compared to male teachers [18,19].

Nevertheless, participants had a better understanding of the symptoms of anaphylaxis. Urticaria was the most recognized symptom, followed by angioedema, facial flushing, and shortness of breath. Less acknowledged symptoms were pruritus, choking, nausea, vomiting, and coma. Factors associated with higher levels of knowledge included female gender, university-level education, and those who had heard of or witnessed a case of anaphylaxis. Regarding our participants' behavior when noticing signs of anaphylaxis, the majority suggested going to the hospital, visiting a primary healthcare facility, or calling an ambulance. Lesser reported actions were avoiding triggers and the use of epinephrine injections. In a different study in the western province of Saudi Arabia, more than half of the population was able to identify symptoms of anaphylaxis. Regarding treatment, only 18% identified the first-line therapy to be epinephrine. The majority were aware that anaphylaxis could be fatal [11,20,21].

Limitations

The study is limited by the use of small sample size and the lack of randomization. Also, there is a weak external validity that interferes with generalization.

## Conclusions

Our study participants demonstrated good knowledge about anaphylaxis triggering factors and a relatively acceptable level of awareness about its symptoms, yet generic knowledge about emergency knowledge of anaphylaxis and the appropriate actions to be taken was poor. More than half did appreciate that anaphylaxis could be fatal. More efforts are needed to increase general public awareness about the seriousness of anaphylaxis and the first aid management steps to be taken. Teachers and families of allergic children need more focused awareness campaigns. Further research is recommended to explore the use of EpiPen and other quick management modalities among teachers and the general population.
